# 4557 Defining the relationship between kidney structure and function in patients with and without diabetes and hypertension

**DOI:** 10.1017/cts.2020.174

**Published:** 2020-07-29

**Authors:** Ghazal Zekavat Quinn, Matthew Palmer, Jordana Cohen, Xin Sheng, Katalin Susztak

**Affiliations:** 1 University of Pennsylvania School of Medicine

## Abstract

OBJECTIVES/GOALS: Histopathological descriptions of kidney tissue provide more information about kidney disease severity and prognosis than serum measures, yet most patients with chronic kidney disease do not undergo kidney biopsy. We aim to develop a method to determine the degree of renal injury in patients with diabetes and hypertension without the need for biopsy. METHODS/STUDY POPULATION: Clinical data and renal tissue samples were collected from 864 patients undergoing tumor-associated nephrectomy in seven medical centers in the United States. Exclusion criteria included age < 18, presence of pyelonephritis or non-diabetic or hypertensive renal disease or incomplete clinical or histopathologic data. 19 histologic parameters were scored in a blinded manner by one renal pathologist. We examined the relationship between and functional variables (such as estimated glomerular filtration rate (eGFR)). Polynomial regression analysis was performed to model histopathologic variables and important clinical parameters such as eGFR RESULTS/ANTICIPATED RESULTS: 607 samples met inclusion criteria and were stratified as: control (no history of diabetes or hypertension, n = 160), hypertension alone (n = 224) and both diabetes and hypertension (n = 223). Interstitial fibrosis (IF) and glomerulosclerosis (GS) had the strongest correlations with eGFR. Regression analysis was used to model histopathologic score for a given eGFR. We found that diabetes and hypertension modified the relationship between tubulointerstitial fibrosis and eGFR. For example, while hypertensive patients without diabetes had 33% IF at an eGFR of 30 ml/min/1.73m^2^ (r^2^ = 0.64, p<0.01), hypertensive patients with diabetes had 32% IF at an eGFR of 30 ml/min/1.73m^2^ (r^2^ = .43, p<0.01) and control patients had approximately 23% IF at an eGFR of 30 ml/min/1.73m^2^ (r^2^ = 0.22, p<0.01). DISCUSSION/SIGNIFICANCE OF IMPACT: Here, we describe the relationship between renal structural changes and renal function and show that hypertension significantly modifies the relationship at a given eGFR. These data can be used to reasonably predict renal structural changes given clinical information without the need for renal biopsy and may provide prognostic value.

